# Precision and accuracy of pre-surgical planning of non-cemented total hip replacement with calibrated digital images and acetates

**DOI:** 10.1186/s13018-021-02584-2

**Published:** 2021-07-03

**Authors:** Luis Fernando Useche Gómez, Hernando Gaitán-Lee, María Alejandra Duarte, Patrick Dennis Halley, Alejandro Romero Jaramillo, Efraim Leal García

**Affiliations:** Pontificia Universidad Javeriana, Hospital Universitario San Ignacio, Cra. 7, No, 40-62 Bogotá, Colombia

**Keywords:** Digital templating, Analog templating, Preoperative planning, Total hip arthroplasty, Total hip replacement, Reliability

## Abstract

**Background:**

When approaching a joint replacement procedure, pre-surgical planning is essential to predict an accurate estimation of implant size and position. There are currently two methods to achieve it, analog and digital. The present study aims to demonstrate how the hybrid technique is accurate and precise for pre-surgical planning in a non-cemented total hip replacement.

**Methods:**

Concordance-type study is used against a gold standard, as well as inter- and intra-observer consistency evaluation of two orthopedic surgeons and two orthopedic surgery residents. Accuracy was calculated with the intra-class correlation coefficient (ICC). Afterwards, the same calculation was done considering a margin of error with one size more and one less.

**Results:**

Thirty-eight patients were included in the study: 19 women and 19 men. Twenty-two prostheses (57.89%) were right-sided and 16 were left (42.11%). Twelve prostheses (31.57%) were Stryker and 26 Johnson & Johnson (68.43%). Acetabular cup correlation compared with the gold standard was moderate: ICC reported 0.45 (95% CI, 0.15–0.76). When adjusted by ± 1 size, ICC was 0.48 (95% CI, 0.18–0.79). On the other hand, results from the femoral stem reported ICC 0.85 (95% CI, 0.07–0.98). When adjusted by ± 1 size, ICC was 0.86 (95% CI, 0.06–0.99).

**Conclusions:**

Hybrid templating is a reliable substitute for analog or digital planning. It is quick, inexpensive, accurate, and better results are observed in the femoral component regardless the level of expertise of the evaluator.

**Level of evidence:**

Grade IV

## Background

Pre-surgical planning in joint replacements allows a size and position estimate of the potential implant. Studies show that planning increases the success and survival rate on the procedure [1], decreasing surgical time [1, 2], instability, limb length discrepancy, periprosthetic fractures, and bone loss [3–7]. An additional advantage is the prevention of requiring implants that are not standard or not available at the time of surgery [7].

Historically, pre-surgical planning has been achieved using printed radiographs [8, 9]. However, this technique has become less popular given its environmental impact and low availability in various clinical settings [10]. The accuracy of this method has been reported to be near 50% for both the acetabular and the femoral components [8, 9]. Currently, most institutions use digital systems to manage radiographic information given the technological advances that allow planning in a digital format with specialized programs [7, 11]. Nonetheless, software used in digital planning is not globally available and high costs restrict its access even further, which is why it is essential to find a planning method that does not require the use of printed radiographs or programs for digital measurement that often are not available [12].

In 2015, Petretta et al. described a hybrid technique in which digital radiographs are used for planning by using templates of the prostheses in acetate. This study showed excellent results in terms of precision and accuracy [12].

Based on the available information about the current topic and author experience, it is hypothesized that the hybrid methodology may have better precision and accuracy than that reported with analog methodology.

## Methods

This is a retrospective concordance-type study against a gold standard (surgically implanted prosthesis), as well as inter- and intra-observer consistency evaluation. The population included are patients scheduled for primary total hip replacement in San Ignacio University Hospital between 2018 and 2019, with primary hip osteoarthritis. Exclusion criteria included a history of fractured pelvis or acetabulum, pelvic or femoral osteotomies, or history of a pelvic or femoral tumor. Unsatisfactory radiographs were excluded as well.

All replacements were performed with Accolade 1 femoral stems and Trident acetabular cups (Stryker) or Corail femoral stems and Pinnacle acetabular cups (Johnson & Johnson).

**Fig. 1:**
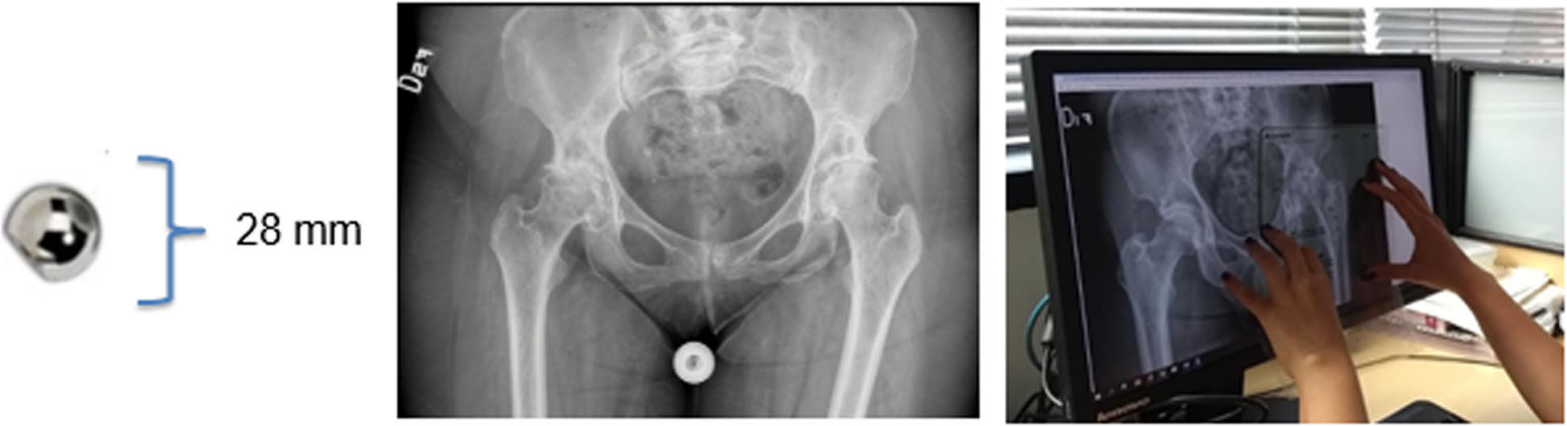
Hybrid planning methodoly

Preoperative radiographs were taken with magnification control to allow the surgeon accurate surgical planning. For this purpose, the tube of the X-ray equipment was placed at a distance of one meter from the patient using a 28-mm diameter metal sphere for magnification control and the pubic-centered beam [13, 14]. The placement indicated for the metallic magnification control was at the level of the femur on the outer or inner side of the thigh for the anteroposterior radiograph, and on the anterior or posterior face of the thigh for the lateral radiography [15–17] (Fig. 1).

The accuracy with the traditional method of pre-surgical planning for the acetabular size has been reported as 52% and for the femoral size 56% [18]. Due to this low performance, the hybrid methodology was used in an attempt to increase precision.

All X-rays were planned by two fourth-year orthopedic surgery residents and two orthopedic surgeons specialized in joint replacements. The planning criteria were those established by De la Valle and Col. In a 15-in. screen, in the Kanteron viewer software, the acetabular cup planning was done. A horizontal reference line was drawn through the base of both teardrops. The ilioischial line, the base of the teardrop, and the superolateral margin of the acetabulum were marked. Then, the acetabular cup template was placed over the 15-in. screen X-ray, with size and location so that, when placed at 40° ± 10° of abduction, its medial border would be in contact with the ilioischial line and have adequate lateral bone coverage. Next, the center of rotation was marked on the radiographs, and compared with the contralateral [19]. The cup size that best suited these parameters was chosen.

Afterwards, the femoral procedure was planned. The limb-length discrepancy was determined by the perpendicular distance from the proximal corner of the lesser trochanter to the reference line. Subsequently, the center of rotation of the femur was marked. The femoral template was placed over the digital X-ray on the screen. Depending on the type of stem, the positioning was performed: For a cementless proximally fitted stem, complete endosteal contact with the lateral and medial cortex of the proximal femur was wanted. Whereas in fully porous-coated stems, complete endosteal contact in the diaphysis was preferred. To modify the length of the limb the template was displaced proximally or distally [19]. The stem size that best suited these parameters was chosen. The size of the acetabular cup and the femoral stem was then recorded. On the day of surgery, details of prosthetic components available in the operating room were recorded as well without making the information available to the operating surgeon so that during the procedure they were blinded to preoperative planning measurements.

The information was collected in a Microsoft Excel spreadsheet based on the review of the records of each patient’s personal medical history that met the inclusion criteria, and data collection was safely archived by the research team, both in digital and physical format.

Sample size was calculated using the R version 3.5.1 program, with an expected agreement of 90%, accuracy of 5% (error), and 95% confidence level; obtaining 38 patients and a total of 304 measurements [20].

**Fig. 2:**
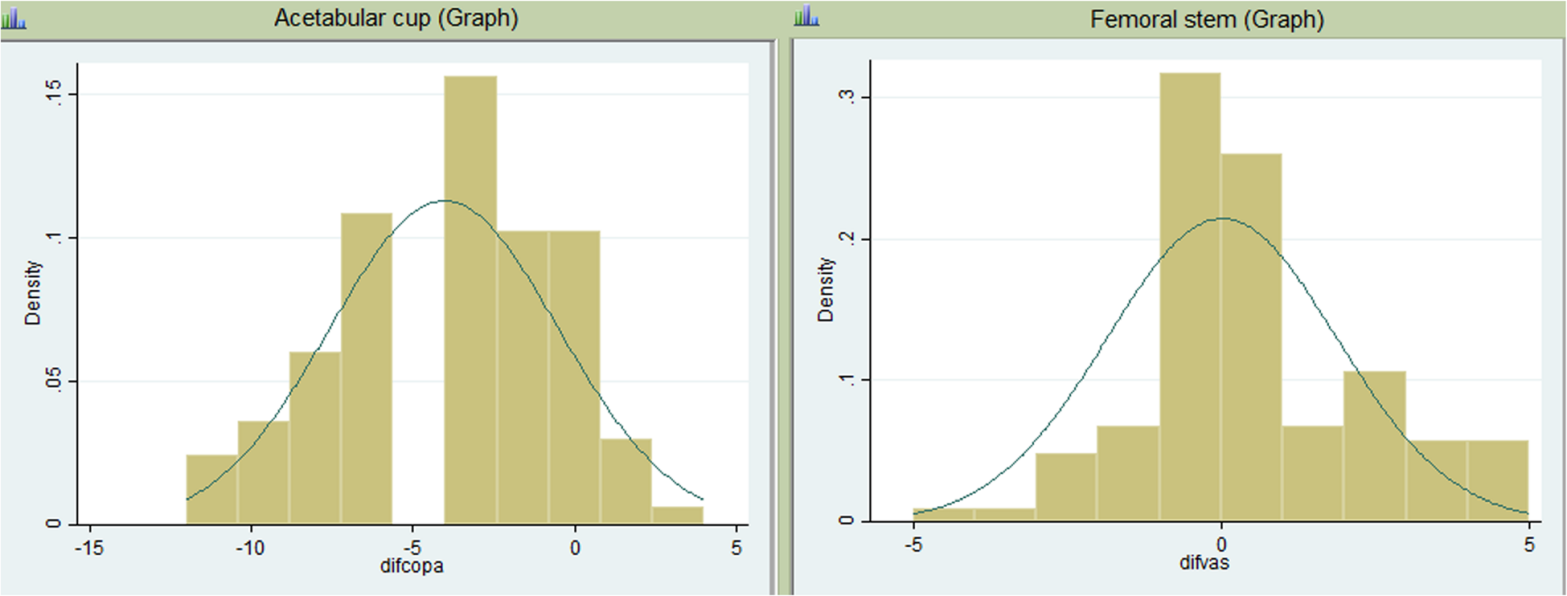
Histograms depicting size difference between the templated and implanted component and their normal distribution curve were generated in STATA

Accuracy measurement was first calculated with the proportion of cases in which the method corresponded exactly to the components, afterward repeating the calculation with a margin of error of 1 size above and 1 size below. Using the same technique as Petretta et al. [12], histograms depicting size difference between the templated and implanted component and their normal distribution curve were generated in STATA (Fig. 2).

Intra-observer and inter-observer reliability were calculated using the intra-class correlation coefficient (ICC). ICC is a method that allows evaluating the general agreement between two or more measurements or observation methods based on an analysis of variance model (ANOVA) with repeated measures. Values below 0.4 indicate low reliability, between 0.4 and 0.75 between fair and good reliability, and values above 0.75 indicate excellent reliability [21]. All statistical analyses were recorded and calculated with STATA program version 13.0.

The work was presented and approved by the ethics committee of the San Ignacio University Hospital and the Pontificia Universidad Javeriana. Given that no additional intervention was performed on patients and the evaluated X-ray images did not allow their identification (nor would other personal patient information would be published in the document), the ethics committee considered that informed consent was not required. All methods were carried out in accordance with relevant international and Colombian guidelines and regulations

## Results

Thirty-eight patients were included in the study: 19 women and 19 men. Twenty-two prostheses (57.89%) were right-sided and 16 were left (42.11%). Twelve prostheses (31.57%) were Stryker and 26 Johnson & Johnson (68.43%).

Acetabular cup correlation compared with the gold standard was moderate: ICC reported 0.45 (95% CI, 0.15–0.76). When adjusted by ± 1 size ICC was 0.48 (95% CI, 0.18–0.79). On the other hand, results from the femoral stem reported ICC 0.85 (95% CI, 0.07–0.98). When adjusted by ± 1 size, ICC was 0.86 (95% CI, 0.06–0.99). The results are summarized in Table 1.

**Table 1:** Acetabular cup and femoral stem correlation compared with the gold standard

	Acetabular cup	Acetabular cup ± 1
ICC	0.45	0.48
	Femoral stem	Femoral stem ± 1
ICC	0.85	0.86

An independent analysis of both the acetabular cup and the femoral stem was carried out for each observer, which is summarized in Table 2.

**Table 2:** Independent analysis of both the acetabular cup and the femoral stem

ICC	Acetabular cup	Acetabular cup ± 1
Evaluator 1	0.62	0.70
Evaluator 2	0.65	0.69
Evaluator 3	0.27	0.31
Evaluator 4	0.67	0.63
ICC	Femoral stem	Femoral stem ± 1
Evaluator 1	0.86	0.86
Evaluator 2	0.82	0.82
Evaluator 3	0.91	0.92
Evaluator 4	0.85	0.85

Evaluators 1 and 3 were orthopedic surgeons specialized in joint replacements. Evaluator 1 acetabular cup ICC was 0.62 (95% CI, 0.17–0.95). When adjusted by ± 1 size, ICC was 0.70 (95% CI, 0.15–0.99). Evaluator 3 acetabular cup ICC was 0.27 (95% CI, 0.2–0.65). When adjusted by ± 1 size ICC was 0.31 (95% CI, 0.22–0.70). Evaluator 1 femoral stem ICC was 0.86 (95% CI, 0.07–1.00). When adjusted by ± 1 size, ICC was 0.86 (95% CI, 0.073–1.00). Evaluator 3 femoral stem ICC was 0.91 (95% CI, 0.05–1.00). When adjusted by ± 1 size, ICC was 0.92 (95% CI, 0.046–1.00).

Evaluators 2 and 4 were fourth year orthopedic surgery residents. Evaluator 2 acetabular cup ICC was 0.65 (95% CI, 0.16–0.96). When adjusted by ± 1 size, ICC was 0.69 (95% CI, 0.15–0.98). Evaluator 4 acetabular cup ICC was 0.63 (95% CI, 0.16–0.97). When adjusted by ± 1 size, ICC was 0.67 (95% CI, 0.17–0.96). Evaluator 2 femoral stem ICC was 0.82 (95% CI, 0.09–0.99). When adjusted by ± 1 size, ICC was 0.82 (95% CI, 0.08–0.99). Evaluator 4 femoral stem ICC was 0.85 (95% CI, 0.07–1.00). When adjusted by ± 1 size, ICC was 0.85 (95% CI, 0.08–1.00).

## Discussion

Traditional methods for the pre-surgical hip replacement procedure planning are performed using a physical X-ray with a 10 to 20% magnification [8, 9]. Two types of templates are included for planning: the acetabular cup and the femoral component. The profiles drawn on the transparent templates overlap the hip radiographs where the anatomical segments are found until the size that fits is found [16]. The accuracy for the acetabular size has been reported as 52% and for the femoral size 56% [18].

The emergence of new digital technologies has decreased the use of physical radiographs, altering the traditional way of pre-surgical planning for joint replacements [11, 18]. Currently, there are multiple available software that allow digital planning; however, most are high cost, which is a common obstacle in the context of a developing country. Available literature has reported an accuracy of 38% of the acetabular component and 35% of the femoral component using this method [22].

Hybrid planning was initially described in 2006 for total hip replacements, consisting of measuring digital images on a liquid crystal monitor to scale so that the image magnifier is the same size as the metric system of the templates used for planning. After this, traditional planning is carried out with acetates [23].

In 2015, Petretta et al. found an accuracy of 77% and 75% in the planning of the acetabular and femoral component respectively. Their study included 260 measurements made by 5 different individuals with different levels of expertise using radiographs of 52 patients. They found no inter- or intra-observer differences and when compared against digital planning. Additionally, it was superior in the planning of the femoral component compared with the digital method, without differences in the acetabular cup [12]. Furthermore, Wang et al., in 2017, report ICC for acetabular templating of 0.918 and ICC for femoral component templating of 0.944 [24].

Considering socio-economical context in a developing country university hospital, author team desired a highly reliable and effective method which was affordable and achieved the best clinical results for patients.

The presented study shows that preparatory planning with the hybrid method has adequate accuracy when compared to the prosthesis that was finally implanted. The femoral component presented in all measurements achieved a correlation near to 0.9 (even without adjustment of sizes) which is interpreted as excellent [25]. Similarly, no differences were found concerning the level of experience or training of the physician who made the measurements. This accuracy was so high, that the possibility of not requiring the margin of error one size above or below in the clinical setting could be considered.

On the other hand, the accuracy of the acetabular component was moderate in all measurements (around 0.45) [25]. However, in this case, having one size above or one below did not significantly affect the accuracy. A probable explanation for lower reliability is that acetabulum is a tridimensional structure with an ellipse shape, which hinders the measures more than a circumference structure, such as the femur diaphysis, especially when taking in to account the limitation that plain radiographs with only two dimensions partakes. Results suggest that in clinical practice, the possible requirement for one or two sizes above or below the size estimated should be anticipated and ordered before entering surgery.

It is important to mention that when an independent analysis between observers was made, significant differences were noticed (Table 2). This was especially important in the acetabular cup measurement of evaluator 3 which had very low ICC, even with the size correction the discrepancy in relation to the others was significant. When we performed a retrospective analysis, we found that this evaluator had an error in the calibration of the measurements in a systematic way that could explain his inferior results.

These results support the author team’s hypothesis that the hybrid technique allows a more accurate pre-surgical planning performance compared to the analog method. The main importance of this finding is that it does not impose additional cost, nor does it require the use of additional costly planning software or applications.

Additionally, this method shows a high correlation between the different evaluators regardless their level of expertise and training. This is extremely important because it shows its effective application in the context of a university hospital.

## Conclusions

The hybrid method is effective for planning total non-cemented primary hip replacements. This technique shows high concordance with the implanted prosthesis and a high correlation between the different evaluators regardless of their level of expertise and training, especially in the femoral stem. Additionally, it is an alternative that does not increase treatment costs. Considering the above mentioned, it is an effective, safe, and economical method that can be used in all areas, specifically and most importantly at hospitals in developing countries.

## Data Availability

The datasets used and/or analyzed during the current study are available from the corresponding author on reasonable request.

## Abbreviations

ICC: Intra-class correlation coefficient
THR: Total hip replacement
ANOVA: Analysis of variance model
